# Correlative Method for Diagnosing Gas-Turbine Tribological Systems

**DOI:** 10.3390/s23125738

**Published:** 2023-06-20

**Authors:** Maciej Deliś, Sylwester Kłysz, Radoslaw Przysowa

**Affiliations:** 1 Air Force Institute of Technology (ITWL), ul. Ksiecia Boleslawa 6, 01-494 Warsaw, Poland; 2 Faculty of Technical Science, University of Warmia and Mazury, Oczapowskiego 11, 10-719 Olsztyn, Poland

**Keywords:** wear debris, oil analysis, emission spectroscopy, turboprop, propeller governor, ANOVA, interaction analysis, condition indicator

## Abstract

Lubricated tribosystems such as main-shaft bearings in gas turbines have been successfully diagnosed by oil sampling for many years. In practice, the interpretation of wear debris analysis results can pose a challenge due to the intricate structure of power transmission systems and the varying degrees of sensitivity among test methods. In this work, oil samples acquired from the fleet of M601T turboprop engines were tested with optical emission spectrometry and analyzed with a correlative model. Customized alarm limits were determined for iron by binning aluminum and zinc concentration into four levels. Two-way analysis of variance (ANOVA) with interaction analysis and post hoc tests was carried out to study the impact of aluminum and zinc concentration on iron concentration. A strong correlation between iron and aluminum, as well as a weaker but still statistically significant correlation between iron and zinc, was observed. When the model was applied to evaluate a selected engine, deviations of iron concentration from the established limits indicated accelerated wear long before the occurrence of critical damage. Thanks to ANOVA, the assessment of engine health was based on a statistically proven correlation between the values of the dependent variable and the classifying factors.

## 1. Introduction

Oil debris monitoring, common in the maintenance of gas turbines, helicopters, and wind turbines, aims to detect wear particles whose characteristics and numbers differ from those generated during normal operation. Monitoring and analyzing oil samples can provide vital information on the health of the engine and predict potential failures, leading to reduced downtime and cost savings. Oil testing has been successfully used to assess the health and ensure the reliability of tribological systems for several decades [1,2,3]. There are many examples in which bearing, transmission, or hydraulic system failures were detected in time. The development of specialized spectrometers [4] made it possible to measure element concentrations with growing precision and process them statistically to set alarm thresholds. However, no oil testing method is universal, and thus various analytic techniques are usually used side by side.

In a traditional offline approach, oil samples are taken from the engine at regular intervals and sent to a laboratory for analysis. Recently, online oil debris monitoring [5,6,7] has become more and more common. It involves installing magnetic [8,9,10], capacitive [11,12], optical [13,14,15], or acoustic sensors [16] in the engine. These sensors can count particles or measure their diameters, but most of them are unable to identify material or detect fine wear debris. Therefore, used oil is still laboratory tested despite the considerable workload and cost related to taking, shipping, and testing samples.

The measured concentrations of elements and other oil parameters are processed statistically to establish alarm thresholds. The traditional approach is based on the normal distribution and assumes triple standard deviation (3σ) to be the warning limit (yellow) and 4σ to be the alarm level (red). If the distribution is not normal, the cumulative distribution is alternatively used to set the warning and alarm limits [17]. In the common data interpretation procedure, level and trend status data from individual test methods are fused to evaluate an overall risk of failure and make maintenance decisions [18,19]. The failure mode matrices are used to link the various concentration level statuses and trend statuses with the specific condition indicator (normal, alert, urgent, hazard, and danger) and potential root causes.

Pair-wise analytical techniques such as correlation [20,21,22] or regression are widespread in oil analysis because relying on a single parameter may not provide reliable solutions and may also overlook significant diagnostic information. Correlation describes a statistical relationship between two random variables. When both variables follow a normal distribution, the Pearson correlation coefficient is used. Otherwise, the Spearman correlation should be employed since it is more general and robust to outliers [23,24]. Other multivariate statistics such as cluster analysis [25], principal component analysis (PCA) [26,27], or factor analysis are also common.

The presented efforts are focused on finding relations and patterns in oil testing results, which are essential for supporting maintenance decisions in gas turbines, helicopters, diesel engines, and wind turbines. However, the interpretation of oil analysis results still poses a challenge when the trends are erratic or individual evaluation methods provide contradictory indications. More efficient oil analysis methods are still being sought to ensure the reliable and safe operation of aircraft engines.

In this work, analysis of variance is used to model the impact of aluminum and zinc concentration on iron concentration and determine alarm limits, which are useful for maintenance decision making. It will be shown that the number of iron particles produced in friction pairs is correlated to aluminum and zinc generation since these elements make up the materials used in the rotating and stationary engine components i.e., steel and aluminum alloys. This correlation provides a basis to set alarm thresholds for iron and detect accelerated wear. The key achievements and contributions of the paper are as follows:

- Studying the correlation between selected metals and finding its physical interpretation.
- Establishing customized iron limits via binning aluminum and zinc concentrations.
- Using two-way ANOVA and interaction analysis for confirming statistical significance of correlations.
- Practical method application for maintenance decision making for two engines.

## 2. Materials and Methods

### 2.1. M601T Turboprop

Walter M601 was developed in the early 1970s, as an alternative to the PT6 turboprop, for the Czech L-410 Turbolet aircraft, a product of Let Kunovice [28,29]. The engine variant M601T, designed a decade later for aerobatic applications, required some design changes, such as the reinforced drive shaft and compressor casing, the modified lubrication system, and others. The engine was produced by Walter Aircraft Engines (now GE Aviation Czech s.r.o.) for the Polish PZL company to power its PZL-130 Orlik TC-I trainer. Its nominal power was 490 kW.

**Figure 1:**
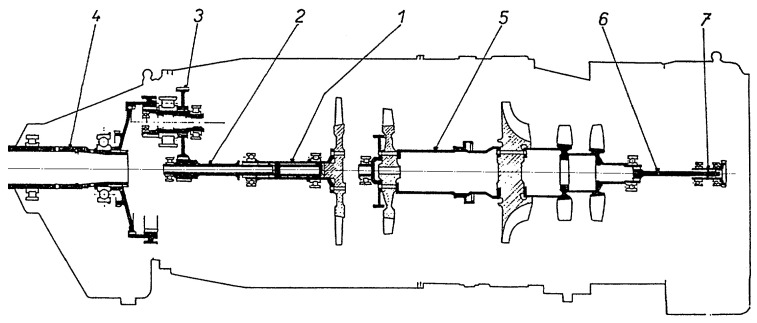
Walter M601 turboprop: 1. Power turbine, 2. connecting shaft, 3. countershaft of reduction unit, 4. propeller shaft, 5. gas generator, 6. elastic shaft, and 7. input drive gear of AGB. Source: [30].

The M601T turboprop pulls the aircraft, so its internal flow is reversed and the air intake is located in its rear part, while there are two elbow exhaust outlets in the front [30,31]. The turboprop consists of two basic sections—a gas generator and a drive unit (Figure 1). The gas generator includes an inlet and a combined compressor–two axial and one centrifugal stage with a total compression of 6.55, an annular combustor, a single-stage generator turbine, an accessory gear box with a fuel control unit, and an electric starter. The drive part of the engine consists of a power (free) single-stage turbine; a two-stage, pseudoplanetary reduction unit; and exhaust outlets. The reduction unit drives the propeller and its governor, and it also supplies the propeller unit with pressurized oil.

The engine oil system includes gear pumps, as well as an integrated oil tank with a capacity of 7 L and a minimum oil level of 5.5 L. The overall amount of oil in the system is 11 L. The nominal oil consumption is 0.1 L per hour [30].

The time between overhaul (TBO) of M601T operated at PZL-130 Orlik was only 500 flight hours (FH). In service, the propeller governor (LUN 7816) turned out to be the weakest part of the M601T engine, and, in the 1990s and early 2000s, its accelerated wear contributed to some air accidents and incidents of the trainer [32]. Consequently, LUN 7816 had to be serviced every 140 FH by the manufacturer. Oil testing and vibration measurement were carried out for engine health monitoring [33,34]. A magnetic plug in the reduction unit was a primary tool to detect oil debris. Additionally, oil was sampled every 10 FH from the tank, reduction unit, and AGB and tested in an accredited laboratory [35]. Samples were taken after the flight, at the appointed time after engine shutdown to ensure sample homogeneity and eliminate the error caused by the sedimentation of wear products. The applied methodology followed the JOAP procedures [36].

### 2.2. Atomic Emission Spectrometry

Spectrometric oil analysis is a diagnostic maintenance tool used to determine the type and amount of wear metals in lubricating fluid samples [36,37,38]. In rotating disc electrode atomic emission spectroscopy (RDE-AES), the wear products contained in the tested oil are excited by an electric arc between graphite electrodes, and the obtained spectral lines are analyzed. The emitted radiation, after splitting on a prism, falls on a plate that transmits radiation with the wavelengths characteristic of the chemical elements under study [39,40]. The measurement is performed on the emission spectrometer simultaneously for several metallic elements. Concentration is measured in parts per million (ppm), i.e., milligrams per kilogram.

The used spectrometer was SpectrOil M by AMETEK Spectro Scientific [41]. It consists of three main components: the excitation source, the optical system, and the readout system. It has the range of 0–1000 mg/kg for iron, aluminum, and zinc. The measurement uncertainty is 10%. Data evaluation criteria and corresponding concentration limits are defined for several military engines by Volume III of the US Joint Oil Analysis Program (JOAP) manual [42]. For M601T and other engines not covered by the standard, alarm thresholds have to be set individually.

The application of RDE-AES in oil analysis is limited by particle size [43,44,45]. It is assumed that it is effective in analyzing debris no greater than 8–10 µm. Therefore, the indication of abnormal wear by spectrometry should be verified by alternative analytic methods, e.g., ferrography.

There are well-known guidelines for the analysis of spectrometry results [36,46,47]. Detected metal debris can be divided into wear products, oil contaminants, or additives. Certain metallic elements can provide clues concerning the parts being worn, but others only offer a general indication of accelerated degradation. Sometimes even the slightest increase or presence of a specific element can be cause for alarm.

In the engine, there are many sources of particles containing iron and aluminum because they are present in many components. Aluminum is mainly a wear product but it can also come from the environment with silicon as dirt contamination. Iron is the most common metal, so its concentration is higher than other elements even without excessive degradation [48]. The concentration of wear metals slowly grows at a constant rate during normal operation. Zinc is used with copper in brass fittings and galvanized surfaces. Regrettably, the origin of the wear could not be determined for the M601T engine since information about the materials was scarce.

### 2.3. Data Analysis

According to the review by Wakiru et al. [49], statistical methods account for 31% of publications on oil analysis, artificial intelligence (AI) approaches for 37%, model-based approaches for 13%, and hybrid approaches for 19%. Even if AI is the main tool, statistical methods are useful to understand the data, select features, and validate the results. For example, Rodrigues et al. [50] used artificial neural networks (ANN) and principal component analysis to classify the oil condition in a fleet of bus diesel engines. Gajewski and Valis [51] proposed multilayer perceptron and radial basis function neural networks to evaluate oil samples from diesel engines of heavy crawlers. Zhao et al. [52] fused vibration-based features with the relative kurtosis and skewness of the ferrous particle size distribution to feed three machine learning classifiers, i.e., support vector machines (SVM), k-nearest neighbors, and decision tree.

The analysis of variance (ANOVA) method, initiated by Fisher in the 1920s, is a statistical tool to analyze the differences among means [53]. It assesses the impact of the independent classifying factor $x_{j}$ (*j* = 1 ⋯, m) on the distribution of the dependent (explained) variable *y*. The levels of the classifying factor (groups) can be binned values or categories. The method analyzes the significance of differences between the means calculated from observations coming from individual groups. The following assumptions of ANOVA have to be met:

1. The distribution of the dependent variable is normal for each group,
2. Variance between groups is similar.

ANOVA is resistant to minor deviations from the normality of the distributions and to small differences in variances between individual groups. To verify the assumption about the normal distribution of the dependent variable, the following statistical tests were used: Shapiro–Wilk, Jarque–Bera, and Lilliefors. To verify the assumption about the homogeneity of variance, Bartlett’s, Levene’s, or Cochran’s tests were used.

There are numerous applications of ANOVA in various disciplines, including wear or oil condition analysis. Holland et al. [54,55] applied Fourier transform infrared (FT-IR) spectroscopy and ANOVA to assess water contamination in oil. Liu et al. [56] used optical measurement to monitor oil debris and viscosity, while ANOVA was utilized to assess the significance of the model equation. Cetin et al. [57] studied the concentration rate and aggregation behavior of nano-silver added colloidal suspensions on the wear behavior of metallic materials. Tian et al. [58] investigated the degeneration of synovial joints and used ANOVA to evaluate the significance of 32 wear parameters obtained by 3D optical surface characterization. Mason et al. [59] evaluated the bearing spall propagation results with ANOVA. Woma [60] analyzed the wear performance of vegetable oils used as lubricants. Azcarate et al. [61] evaluated D-optimal mixture designs for microwave-induced plasma optical emission spectrometer. ANOVA is also frequently used to analyze metal contaminants in food, drugs, or water with atomic emission spectrometry. However, we could not find any publications in which ANOVA was employed in oil analysis for modeling wear metal concentration.

This study aims to determine whether the concentrations of aluminum and zinc affect the concentration of iron. Therefore, the concentrations of aluminum and zinc are classifying factors, while the dependent (explained) variable is iron concentration.

The following tasks were performed to implement ANOVA for wear debris analysis:

1. Conduct exploratory data analysis to study concentration distributions and understand the data.
2. Run tests for the homogeneity of variance to select the best variant of binning aluminum and zinc data.
3. Bin the data into four Al and Zn levels and remove outliers.
4. Transform iron concentration to normal distribution.
5. Tests the normality of transformed iron distributions in individual groups.
6. Calculate two-way ANOVA.
7. Run post hoc tests.
8. Analyze interaction plots.
9. Use the model to set the individual limits of iron concentration for each factor level.
10. Compare the sample testing results with the defined limits to make a maintenance decision.

## 3. Results

### 3.1. Dataset

The oil samples acquired from the fleet of 29 M601T engines operated over five years were tested using optical emission spectrometry with a rotating electrode. The dataset included 1250 samples with the measured concentrations of 19 elements: Ag, Al, B, Ba, Ca, Cr, Cu, Fe, Mg, Mo, Na, Ni, P, Pb, Si, Sn, Ti, V, and Zn. The elements whose concentration exceeded the threshold of 1 ppm were selected for statistical analysis. In this case, only aluminum, iron, and zinc met this condition, while other elements generally produced negligible values. The observed iron concentrations were the highest because this element is contained in many alloys used in the engine. For this reason, iron was selected as a dependent variable, and its values were used to classify engine health.

**Figure 2:**
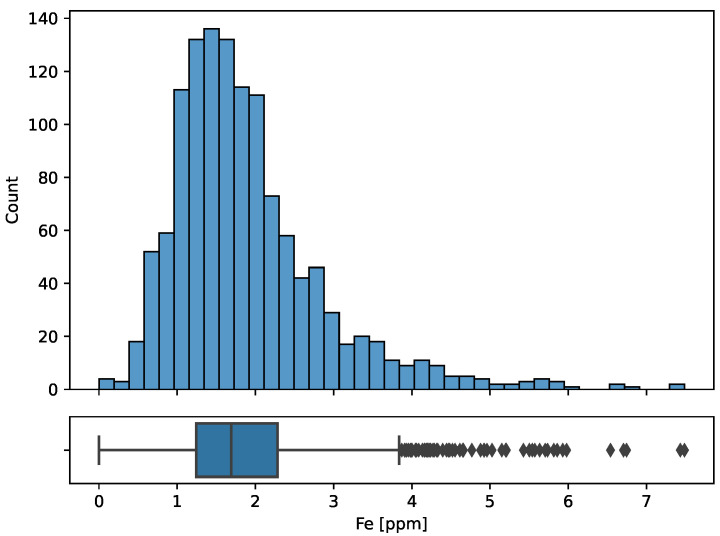
Distribution of iron concentration.

**Figure 3:**
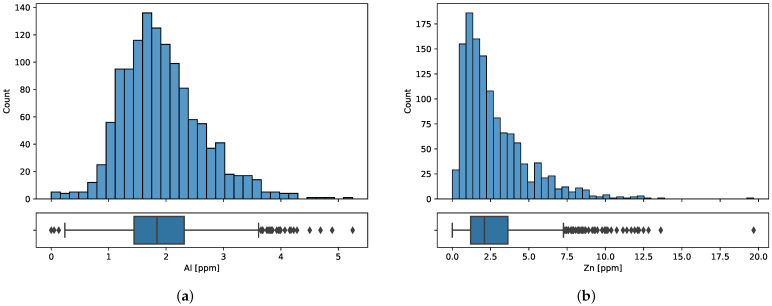
Distributions of (**a**) aluminum and (**b**) zinc concentration.

**Figure 4:**
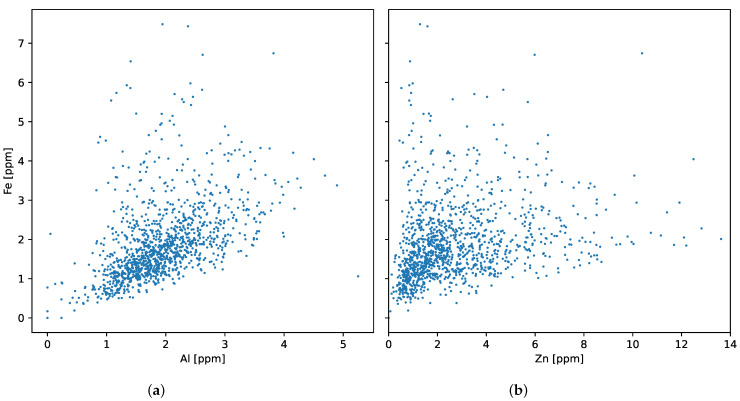
Iron concentration vs. (**a**) aluminum and (**b**) zinc.

The following plots present the distribution of iron (Figure 2), aluminum, and zinc (Figure 3). Iron distribution is close to normal but has a positive skew of 1.68. Scatter plots (Figure 4) confirm that there is a positive correlation between elements that is higher for aluminum than for zinc. The Pearson correlation coefficients are presented in Table 1.

### 3.2. Binning Al and Zn Concentrations

To perform ANOVA, Al and Zn concentrations have to be binned into some groups. Therefore, the range of their observations was divided into a number of equal bins, with the right endpoint chosen to include 99.7% of observations (three-sigma rule). Several binning variants were tested for ANOVA’s assumptions to be met. Therefore, Bartlett’s and Levene’s tests were used to evaluate iron variance homogeneity between individual Al and Zn levels. Outliers (with values greater than Q3 + 1.5 IRQ) were removed before the tests.

Table 2 presents the probability values produced by Bartlett’s and Levene’s tests for the increasing number of aluminum and zinc levels. Probabilities exceeding 0.5 are marked in green. The case of two Al and Zn levels was analyzed further, but it was found that the iron concentration in some of these groups did not pass distribution normality tests (Lilliefors, Jarque–Bera or $\chi^{2}$, Table 3), and, consequently, ANOVA could not be performed.

### 3.3. Transforming Data to Normal Distribution

Since the iron concentration did not meet ANOVA assumptions in any tested binning variant, data transformation was applied using the natural logarithm function:

(1) f(y)=ln(y+b)

**Figure 5:**
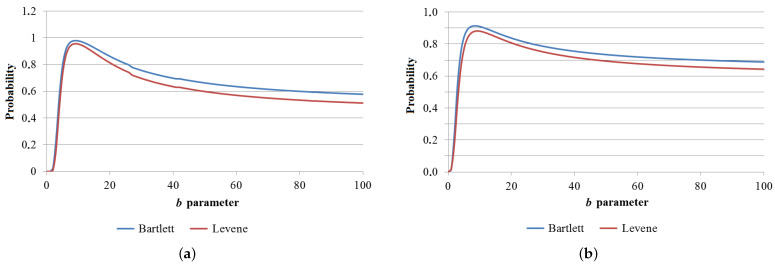
Probability of variance homogeneity as a function of b parameter for four groups: (**a**) Aluminum (**b**) Zinc.

A similar testing procedure was carried out for the transformed data to check the homogeneity of variances between groups and find the best binning variant. The value of parameter *b* was sought iteratively (Figure 5).

Table 4 shows the estimated probability values of Bartlett’s and Levene’s tests for different numbers of classification factors (ranges of aluminum and zinc concentrations) after the logarithmic transformation of the data. The table shows the value of factor *b*, for which the highest probability values for both aluminum and zinc levels were obtained. Green indicates the probability that exceeds the limit of 0.5. The case of four groups and parameter *b* = 9 achieved the highest probability both for aluminum and zinc, so it was selected for further analysis of variance.

**Figure 6:**
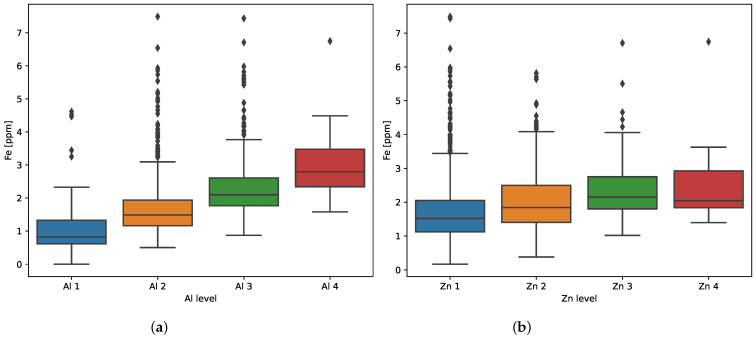
Distributions of iron concentration for individual (**a**) aluminum and (**b**) zinc levels before removing outliers and logarithmic transformation.

The aluminum and zinc concentration levels chosen for binning into four groups are specified in Table 5. The corresponding iron distributions are presented in Figure 6. These box plots show some outliers (with values greater than Q3 + 1.5 IRQ), which were removed from each group before further analysis. Then, the group means and standard deviations (Std) of transformed iron concentration were calculated (Table 5).

Then, the distributions of transformed iron for four levels of aluminum and zinc concentration were tested for normality (Table 6). At least one of three tests suggested accepting the null hypothesis in each case, so the distributions could be considered normal and the assumptions of ANOVA were satisfied (at the 5% significance level).

### 3.4. Two-Way ANOVA

A two-factor analysis of the variance of transformed iron was performed for four levels of aluminum and zinc concentration. The ANOVA table (Table 7) shows the effect of these factors on iron concentration, without testing interactions.

In both cases, comparing the determined values of F (165.42 and 9.91) with its critical values, we decide to reject the null hypothesis about the equality of means in all of the groups in favor of the alternative hypothesis that there is an impact of both aluminum and zinc on iron.

There is a significant difference in the sum of the squares between aluminum (1.063) and zinc (0.056). This indicates that aluminum has a greater impact on iron concentration (27.9%) than zinc (1.5%). This can be confirmed by comparing the mean values of the different groups of aluminum and zinc. The means of aluminum groups varies more from each other than zinc groups (Table 5).

**Figure 7:**
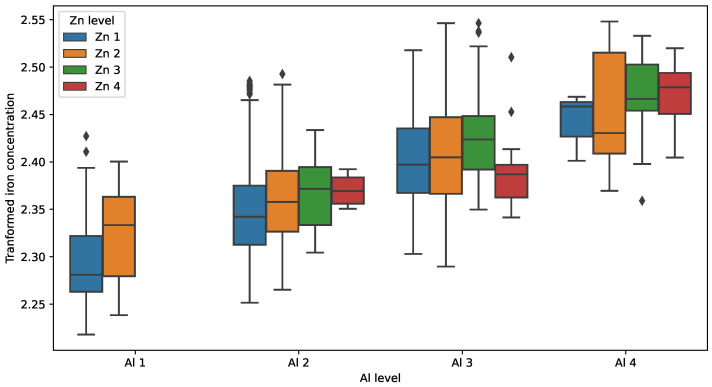
Grouped boxplot of transformed iron concentration.

The attempt to perform ANOVA with testing interaction effects failed due to incomplete data representation, i.e., there were no observations belonging to the paired groups Al 1 Zn 3 and Al 1 Zn 4. This is illustrated by a grouped boxplot in Figure 7. The issue was solved by removing the data from group Al 1, and then ANOVA with interactions was smoothly completed (Table 8). Removing Al 1 did not affect the diagnostic reasoning since it contained only 85 data points with the lowest concentration.

Based on the F test result (Prob > F), the null hypothesis that there are no differences between means in individual groups should be rejected. The table shows that both the concentration of aluminum and zinc affect the results of iron. The effect size shows that the greatest impact has aluminum concentration (22.7%), then zinc concentration (2.1%), and the least interaction between aluminum and zinc (0.5%). However, the impact of the interaction is not statistically significant.

### 3.5. Post Hoc Tests

After receiving ANOVA results confirming statistically significant differences between the means of individual groups, the next step is to perform post hoc tests. Since ANOVA verified only that means in some groups are not equal, these tests are necessary to determine which groups’ means are significantly different. There are several post hoc tests that use different approaches, and some of them are considered conservative. In this work, the least significant differences test (LSD), as well as Bonferroni’s, Scheffé’s and Tukey’s tests, was used.

The results of post hoc tests for aluminum concentration levels are presented in Table A1 and Table A2. All of the performed tests confirmed that the means in all of the groups significantly differ from each other at the 95% confidence level. Their confidence intervals did not include zero for any pair of means analyzed.

The results of the LSD, Bonferroni’s, Scheffé’s, and Tukey’s tests for zinc concentration levels are presented in Table A3 and Table A4. All of the post hoc tests showed that the mean values for Zn concentration levels are significantly different at the 95% confidence level, except the Zn 3 and Zn 4 groups. Since the confidence interval of this pair of levels includes zero (marked in yellow), it can be concluded that the averages of these groups do not differ significantly. This is also confirmed by Figure 6, where the overlapping of Zn 3 and Zn 4 intervals is visible.

For all of the performed tests, zinc compared to aluminum was characterized by the lower absolute values of differences of group means, which is in line with the effect sizes calculated by ANOVA.

### 3.6. Interaction Effects

Interaction plots are a convenient way to illustrate how factors influence iron concentration and further analyze their interactions. These plots have the group means of the dependent variable on the ordinate and the level of one of the factors on the abscissa. The levels of the second factor correspond to individual data series in the plot. The shape of the polylines—their crossing, curving, and parallelism—illustrates the effects of the interaction.

**Figure 8:**
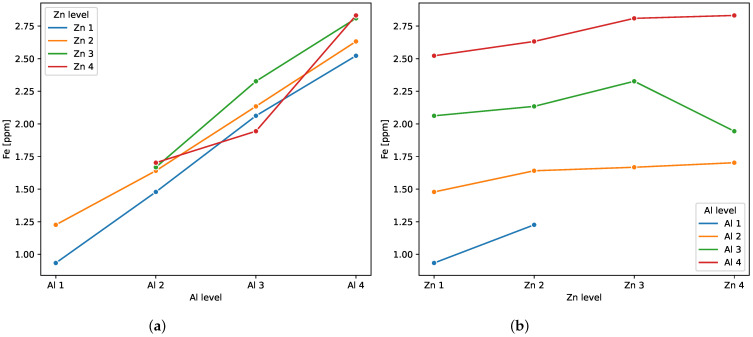
Interaction plot—the concentration of iron vs. (**a**) aluminum and (**b**) zinc level.

Figure 8a shows iron as a function of aluminum levels 2–4. The lines are almost parallel, with a deviation at Al 3 for Zn 4 line. Small distances between the lines indicate that there is a small effect of zinc, while the high slope of the curves confirms a clear effect of aluminum.

Similarly, Figure 8b, with iron versus Zn levels, shows roughly parallel lines with deviations at Zn 4 for Al 3 line. The low slope of the curves confirms that there is a small effect of zinc, while considerable distances between the lines indicate a clear effect from aluminum. The slight deviation from parallelism at two points suggests the existence of weak interaction, i.e., at different levels of aluminum concentration, zinc concentration affects the concentration of iron differently. Interaction plots are in line with effect sizes produced by ANOVA (Table 8), which suggested a significantly higher impact of aluminum than zinc or their interactions.

### 3.7. Setting Condition Indicators

Based on the presented ANOVA results, it can be assumed that for the average M601T engine, during normal operation, the iron concentration for individual aluminum and zinc levels should remain within limits defined by the statistical model (Table 9). These limits corresponded to two-sigma intervals in these groups.

### 3.8. Engine 1

The obtained ANOVA model was used to evaluate the wear of two engines.

**Figure 9:**
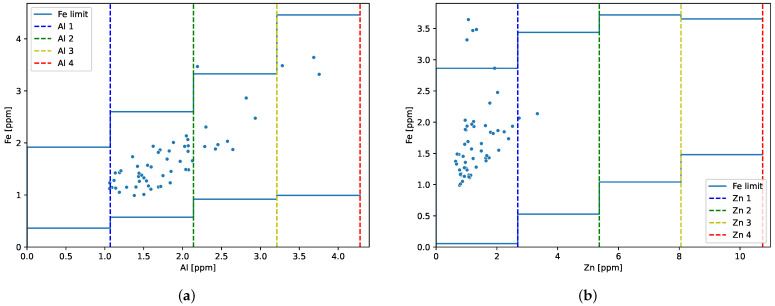
Engine 1—iron concentration vs. (**a**) aluminum or (**b**) zinc concentration with the upper limit of iron for individual groups.

For engine 1, between 697.6 and 1254 FH, oil samples were collected from the tank every 10^±2^ FH (in total 57 samples). The scatter plot (Figure 9) presents the iron results as a function of the aluminum or zinc concentration along with the boundaries of their four levels and the corresponding iron limits. The measurement uncertainty of 10% must be taken into account when classifying the results.

It is noticeable that one iron data point exceeded the upper limit of iron concentration at AL 3. In contrast, as a function of zinc concentration, four iron concentration values exceeded the upper limit of iron concentration at ZN 1.

**Figure 10:**
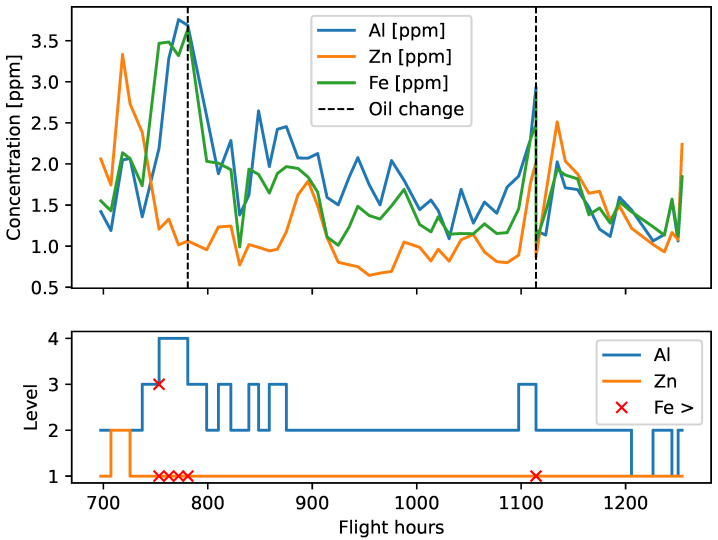
Engine 1—concentration time series.

Figure 10 shows the time series of iron, aluminum, and zinc concentration along with their level number. The moments when the upper limit of iron concentration was exceeded are marked with a cross mark symbol. When iron concentration exceeded the Al-related limit, the zinc-related limit was also exceeded. Three other Zn 1 exceedances are located nearby.

From the maintenance records of the aircraft, it was found that the oil sample taken at 753.33 FH was the first sample taken after the overhaul. In addition, after sampling the oil at 780.9 FH, the oil was changed. Before the overhaul, the upper limit of iron concentration value had not been exceeded. Therefore, the above results indicate that the contamination of the lubrication system after the overhaul distorted the statistically proven correlation between the discussed elements. After the oil change, no further deviations from the calculated correlation were found during the next 450 FH.

### 3.9. Engine 2

**Figure 11:**
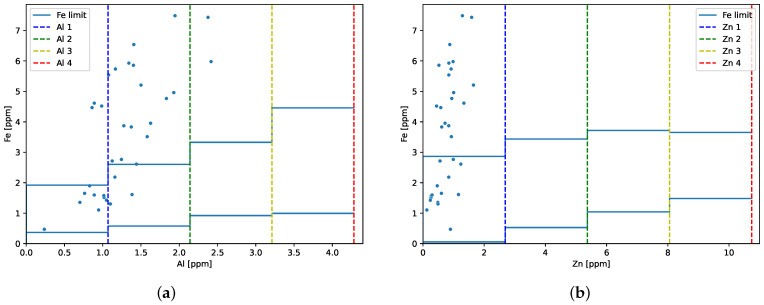
Ironconcentration as a function of (**a**) aluminum or (**b**) zinc concentration with marked values of the upper limits of the levels and the upper values of iron for individual levels.

Engine 2 accumulated 808.08 FH when the first oil sample was taken. Before reaching 1114.38 FH, the 33 oil samples were collected and analyzed. Figure 11 shows the obtained iron results as a function of aluminum or zinc concentration. The plots show that iron concentration exceeded the upper limit for some aluminum and zinc levels.

**Figure 12:**
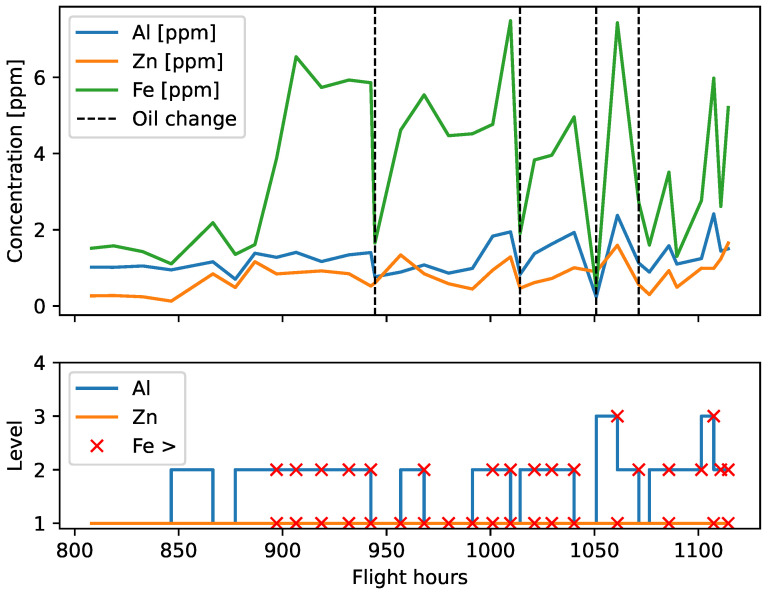
Iron concentration as a function of flight hours.

Figure 12 shows the time series of iron, aluminum, and zinc concentration with their respective level numbers. The moments when the upper limit of iron concentration was exceeded are marked with a cross mark symbol. In the first 80 FH, iron concentration did not cross the limit, but later numerous exceedances were observed. Several oil changes combined with the flushing of the system led to a temporary reduction in particle concentration and the acquisition of some samples that were within the limits. However, these actions did not reverse the overall trend of growing iron production.

The traditional analytic approach (3σ) classified the observed iron results 5.5–7.5 ppm as acceptable in the four-point scale: (1) white—undamaged or new, (2) green—acceptable wear, (3) yellow—increased wear, and (4) red—significant deterioration. It can be concluded that although iron concentration was only at the yellow level, the ANOVA-based model indicated the accelerated wear of the system. Already 215 FH before detecting unacceptable particles at the magnetic chip detector, symptoms of damage appeared in the form of a distorted correlation between the concentration of the discussed elements. Even repeated oil changes and other maintenance actions such as replacing the propeller governor did not result in a permanent return to the correct correlation of results.

**Figure 13:**
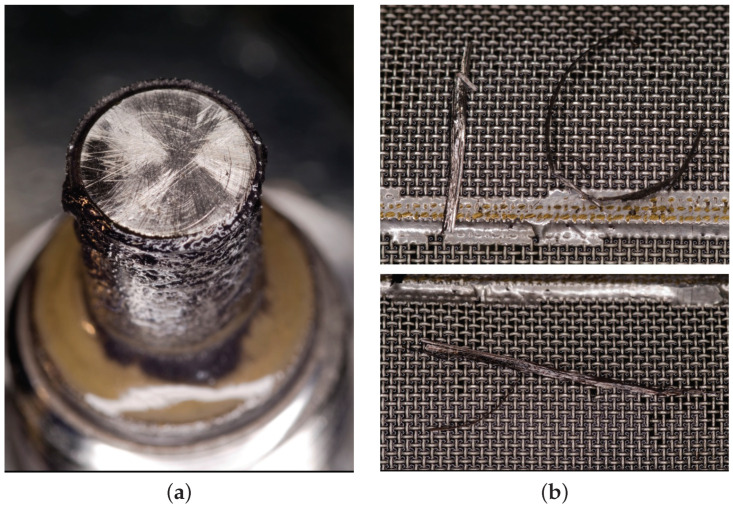
Engine 2: (**a**) numerous metallic particles on the magnetic plug of AGB; (**b**) hairy chips up to 3 cm long on the filter of the propeller reduction unit.

The process of accelerated wear distorted the statistical dependence between the elements because it triggered the generation of particles in larger quantities and with an unusual distribution. Since iron concentration persistently exceeded the limits for some Al an Zn levels, the engine was subjected to supervised maintenance, which involved more frequent check-ups. This procedure made it possible to continue engine operation safely and avoid the costs of a premature overhaul. However, upon reaching 1114 FH, hairy chips up to 3 cm long were found on the filter of the propeller reduction unit along with numerous metallic particles on the magnetic plug of AGB (Figure 13). The engine was grounded and sent to the repair shop.

## 4. Discussion

ANOVA was employed here to prove the statistical dependence between iron and aluminum or zinc and to calculate the effect size for each factor. Its results led us to reject the null hypothesis of mean equality in individual groups. This confirms that with the increase in the concentration of aluminum and zinc, higher average values of iron concentration are obtained (except Zn 4 level). Therefore, their concentrations should be analyzed jointly in this tribological system (the M601T turboprop) since their individual monitoring is less effective. For example, an increase in the concentration of aluminum without an increase in the concentration of iron, or vice versa, exceeding the established group limits may indicate a change in the wear process leading to damage. These correlations can be interpreted as footprints of the alloys containing the analyzed elements, forming friction pairs in the engine. However, without the material map of the engine, the only way to find the root cause of accelerated wear is to tear it down and inspect the components in a material laboratory.

It should be noted that although the impact of zinc concentration was found to be weaker than the impact of aluminum, its effect was considered statistically significant by ANOVA and should be taken into account. It is likely that in other systems, the influence of other elements can be neglected, and it will be sufficient to monitor only the pair of iron and aluminum. However, it cannot be excluded that in some cases there will be more than two elements that significantly affect iron concentration, but the proposed approach can be still used then.

Although the proposed method was applied to the M601T turboprop, it is general and independent of the engine type. A similar approach is used by ITWL for other platforms operated by the Polish Armed Forces. Friction pairs producing iron and aluminum particles exist in all aviation engines. Durability problems with propeller governors are quite common in military or high-performance aircraft with intensive or aggressive mission profiles. While T56 and PT6 turboprops are covered by JOAP, many other engines are not. Therefore, lessons learned from this case study can be particularly useful to operators who implement an oil analysis procedure for a less popular engine.

Binning aluminum and zinc concentration into four levels simplified the classification problem since it converted continuous variables into categorical ones. This allowed fixed alarm limits to be found for each group. When compared to other more advanced methods, these thresholds are more convenient to use in practice. Additionally, they are based on individual group means and thus describe the system better than linear regression, where the model relies on a single mean. However, selecting the right number of levels and their boundaries to meet ANOVA conditions can sometimes be challenging. By employing factor variables, this work expands on prior research based on the pair-wise analysis of oil parameters [15,24,62].

Obviously, ANOVA is a linear model and has its limitations. Some issues, related to skewed data distribution, were overcome here by removing outliers and logarithmic transformation. Undoubtedly, this dataset and similar oil analysis results can be processed with more advanced statistical or machine learning models such as quantile regression, decision trees [63,64], SVM [65,66], artificial neural networks [67], or their assemblies [68,69]. There are enough training data, and the computational cost is moderate, but the main challenge is to properly formulate the prediction problem when engine wear and its remaining useful life is not a priori known. Consequently, classical statistical methods, due to their convenience, remain important in maintenance decision making.

## 5. Conclusions

It was shown that an ANOVA-based model can be used in practice to model the wear products in oil samples. It provided valuable diagnostic information for data that, analyzed with traditional single-parameter analysis, did not exhibit significant deviations from normal wear. Deviations of iron concentration from the limits determined by the statistical model may indicate the accelerated wear of the engine long before the occurrence of critical damage or high oil contamination by wear products.

The most important finding of the work was that the strong correlation of iron and aluminum, as well as the weaker but still statistically significant correlation of iron and zinc, was confirmed. These correlations were most likely related to the composition of alloys that form friction pairs in the engine. Consequently, the correlative model outperformed the traditional approach in which metal concentrations are individually evaluated. By providing customized alarm limits, it could be directly implemented in practice to diagnose gas-turbine engines.

There is clearly a need for more research in this area. Future studies should incorporate data from other oil analytic methods and explore the proposed approach in diverse tribological systems such as the main and rear gearboxes of helicopters. This will advance our understanding of wear mechanisms, improve predictive models, and ultimately pave the way for more efficient and robust tribological systems in various industrial sectors.

## Figures and Tables

**Table 1:** Pearson correlation coefficients.

	Fe [ppm]	Al [ppm]	Zn [ppm]
Fe [ppm]	1.00	0.46	0.25
Al [ppm]	0.46	1.00	0.53
Zn [ppm]	0.25	0.53	1.00

**Table 2:** Bartlett’s and Levene’s probabilities for the increasing number of groups.

	Number of Groups								
Test	2	3	4	5	6	7	8	9	10
Al Bartlett	0.6068	0.0030	0.4883	0.0002	0.0000	0.0000	0.0000	0.0000	0.0000
Al Levene	0.5458	0.0009	0.4244	0.0000	0.0000	0.0001	0.0000	0.0004	0.0000
Zn Bartlett	0.9254	0.4547	0.6357	0.1212	0.0024	0.0000	0.0017	0.0000	0.0000
Zn Levene	0.9871	0.4159	0.5858	0.0536	0.0001	0.0000	0.0004	0.0000	0.0000

**Table 3:** Distribution normality tests for two aluminum and zinc levels.

Group	Test	Result	Significance
	Lillieforsa	1	0.001
Al 1	Jarque–Bera	1	0.001
	$\chi^{2}$	1	0
	Lillieforsa	0	0.062
Al 2	Jarque–Bera	0	0.1341
	$\chi^{2}$	0	0.0554
	Lillieforsa	1	0.001
Zn 1	Jarque–Bera	1	0.001
	$\chi^{2}$	1	0
	Lillieforsa	1	0.0122
Zn 2	Jarque–Bera	0	0.0655
	$\chi^{2}$	0	0.1512

**Table 4:** Bartlett’s and Levene’s probabilities for transformed data.

	Number of Groups								
Test	2	3	4	5	6	7	8	9	10
Al Bartlett	0.5621	0.5785	0.9791	0.2841	0.4005	0.4583	0.5724	0.0168	0.0542
Al Levene	0.5005	0.4648	0.9563	0.1432	0.2624	0.3440	0.3065	0.2847	0.0012
Zn Bartlett	0.9582	0.5488	0.9132	0.8641	0.0227	0.0069	0.0608	0.0006	0.0010
Zn Levene	0.9758	0.4472	0.8814	0.8177	0.0082	0.0061	0.0293	0.0005	0.0001
*b* parameter	170.0	3.5	9.0	5.0	2.0	1.5	1.5	1.5	1.0

**Table 5:** Defined aluminum and zinc levels and corresponding transformed iron values.

	Boundaries (ppm)		Observations		Transformed Fe	
Group	Left	Right	Count	Outliers	Mean	Std
Al 1	0.0000	1.0704	90	5	2.29693	0.0468
Al 2	1.0704	2.1408	741	48	2.35268	0.0492
Al 3	2.1408	3.2112	348	24	2.40928	0.0513
Al 4	3.2112	4.2816	64	1	2.47459	0.0625
Zn 1	0.0000	2.6856	779	48	2.35518	0.0591
Zn 2	2.6856	5.3712	321	14	2.38994	0.0654
Zn 3	5.3712	8.0569	108	5	2.41880	0.0621
Zn 4	8.0569	10.7425	31	1	2.41990	0.0590

**Table 6:** Tests for distribution normality of transformed iron data for individual Al and Zn levels.

Test	Group	Result	Significance	Group	Result	Significance
Lillieforsa		1	0.0010		0	0.0520
Jarque–Bera	Al 1	0	0.0708	Zn 1	1	0.0068
$\chi^{2}$		1	0.0023		1	0.0003
Lillieforsa		1	0.0010		1	0.0451
Jarque–Bera	Al 2	0	0.0511	Zn 2	1	0.0425
$\chi^{2}$		1	0.0005		0	0.0641
Lillieforsa		0	0.1665		0	0.3512
Jarque–Bera	Al 3	0	0.0793	Zn 3	0	0.2220
$\chi^{2}$		0	0.3232		0	0.3341
Lillieforsa		0	0.3315		0	0.2529
Jarque–Bera	Al 4	0	0.4094	Zn 4	0	0.1572
$\chi^{2}$		0	0.3141		1	0.0110

**Table 7:** Two-way ANOVA without interaction effects.

Source	Sum Sq.	d.f.	F	Prob > F	Effect Size
Al	1.063465	3	149.088665	1.885264 $\times \;10^{-81}$	0.278545
Zn	0.055779	3	7.819726	3.615759 $\times \;10^{-5}$	0.014610
Residual	2.698691	1135			

**Table 8:** Two-way ANOVA with interactions after removing Al 1 level.

Source	Sum Sq.	d.f.	F	Prob > F	Effect Size
Al	0.734975	2	153.271682	4.261817 $\times 10^{-59}$	0.226810
Zn	0.052767	3	7.335961	7.214323 $\times 10^{-5}$	0.020626
Al*Zn	0.012550	6	0.872404	0.5145026	0.004984
Residual	2.505514	1045			

Note: The star symbol in Al*Zn denotes interaction between Al and Zn.

**Table 9:** Concentration of aluminum and zinc and the corresponding iron concentration limits.

	Lower Limit	Upper Limit	Fe Lower Limit	Fe Upper Limit
Interval	(ppm)	(ppm)	(ppm)	(ppm)
Al 1	0.0000	1.0704	0.0559	1.9183
Al 2	1.0704	2.1408	0.5279	2.6015
Al 3	2.1408	3.2112	1.0416	3.3275
Al 4	3.2112	4.2816	1.4807	4.4589
Zn 1	0.0000	2.6856	0.3650	2.8625
Zn 2	2.6856	5.3712	0.5748	3.4378
Zn 3	5.3712	8.0569	0.9207	3.7174
Zn 4	8.0569	10.7425	0.9933	3.6528

## Data Availability

Restrictions apply to the availability of the source data. Upon written request, access can be granted by ITWL.

## Abbreviations

The following abbreviations are used in this manuscript:

AI	Artificial intelligence
Al	Aluminum
AGB	Accessory gear box
ANN	Artificial neural networks
ANOVA	Analysis of variance
CI	Confidence interval
d.f.	Degree of freedom
Fe	Iron
FH	Flight hour
FT-IR	Fourier transform infrared spectroscopy
IRQ	Interquartile range
ITWL	Air Force Institute of Technology in Warsaw
JOAP	Joint Oil Analysis Program
LSD	Least significant difference test
ODM	Oil debris monitoring
PCA	Principal component analysis
ppm	Parts per million (mg/kg)
RDE-AES	Rotating disc electrode atomic emission spectroscopy
Std	Standard deviation
SVM	Support vector machine
TBO	Time between overhaul
Zn	Zinc

## Appendix A. Results of Post Hoc Tests

**Table A1:** LSD and Bonferroni’s test results and their confidence intervals (CI) for Al concentration levels.

Compared		CI Left	LSD Test	CI Right	CI Left	Bonferroni	CI Right
Groups		Boundary	Result	Boundary	Boundary	Result	Boundary
Al 1	Al 2	−0.0690	−0.0588	−0.0486	−0.0725	−0.0588	−0.0451
Al 1	Al 3	−0.1239	−0.1130	−0.1022	−0.1276	−0.1130	−0.0984
Al 1	Al 4	−0.1790	−0.1623	−0.1457	−0.1848	−0.1623	−0.1399
Al 2	Al 3	−0.0604	−0.0542	−0.0481	−0.0625	−0.0542	−0.0459
Al 2	Al 4	−0.1176	−0.1035	−0.0895	−0.1225	−0.1035	−0.0846
Al 3	Al 4	−0.0639	−0.0493	−0.0348	−0.0689	−0.0493	−0.0297

**Table A2:** Scheffe’s and Tukey’s test results and their confidence intervals (CI) for Al concentration levels.

Compared		CI Left	Scheffe	CI Right	CI Left	Tukey	CI Right
Groups		Boundary	Result	Boundary	Boundary	Result	Boundary
Al 1	Al 2	−0.0733	−0.0588	−0.0443	−0.0721	−0.0588	−0.0455
Al 1	Al 3	−0.1285	−0.1130	−0.0975	−0.1272	−0.1130	−0.0988
Al 1	Al 4	−0.1861	−0.1623	−0.1386	−0.1841	−0.1623	−0.1405
Al 2	Al 3	−0.0630	−0.0542	−0.0454	−0.0623	−0.0542	−0.0461
Al 2	Al 4	−0.1236	−0.1035	−0.0835	−0.1219	−0.1035	−0.0851
Al 3	Al 4	−0.0701	−0.0493	−0.0286	−0.0684	−0.0493	−0.0303

**Table A3:** LSD and Bonferroni’s test results and their confidence intervals (CI) for Zn concentration levels.

Compared		CI Left	LSD Test	CI Right	CI Left	Bonferroni	CI Right
Groups		Boundary	Result	Boundary	Boundary	Result	Boundary
Zn 1	Zn 2	−0.0380	−0.0305	−0.0231	−0.0406	−0.0305	−0.0205
Zn 1	Zn 3	−0.0706	−0.0590	−0.0474	−0.0746	−0.0590	−0.0434
Zn 1	Zn 4	−0.0868	−0.0667	−0.0465	−0.0937	−0.0667	−0.0396
Zn 2	Zn 3	−0.0410	−0.0285	−0.0159	−0.0454	−0.0285	−0.0115
Zn 2	Zn 4	−0.0568	−0.0361	−0.0154	−0.0640	−0.0361	−0.0082
Zn 3	Zn 4	−0.0302	−0.0076	0.0149	−0.0380	−0.0076	0.0227

**Table A4:** Scheffe’s and Tukey’s test results and their confidence intervals (CI) for Zn concentration levels.

Compared		CI Left	Scheffé	CI Right	CI Left	Tukey	CI Right
Groups		Boundary	Result	Boundary	Boundary	Result	Boundary
Zn 1	Zn 2	−0.0412	−0.0305	−0.0199	−0.0403	−0.0305	−0.0208
Zn 1	Zn 3	−0.0756	−0.0590	−0.0425	−0.0742	−0.0590	−0.0438
Zn 1	Zn 4	−0.0954	−0.0667	−0.0380	−0.0930	−0.0667	−0.0403
Zn 2	Zn 3	−0.0464	−0.0285	−0.0105	−0.0449	−0.0285	−0.0120
Zn 2	Zn 4	−0.0656	−0.0361	−0.0066	−0.0632	−0.0361	−0.0090
Zn 3	Zn 4	−0.0398	−0.0076	0.0245	−0.0371	−0.0076	0.0218
